# Direct Ink Writing of Recyclable Supramolecular Soft Actuators

**DOI:** 10.1021/acsmacrolett.2c00359

**Published:** 2022-07-08

**Authors:** Sean J. D. Lugger, Ruth M. C. Verbroekken, Dirk J. Mulder, Albert P. H. J. Schenning

**Affiliations:** † Laboratory of Stimuli-Responsive Functional Materials and Devices (SFD), Department of Chemical Engineering and Chemistry, Eindhoven University of Technology (TU/e), P.O. Box 513, 5600 MB Eindhoven, The Netherlands; ‡ Institute for Complex Molecular Systems, Eindhoven University of Technology (TU/e), P.O. Box 513, 5600 MB Eindhoven, The Netherlands

## Abstract

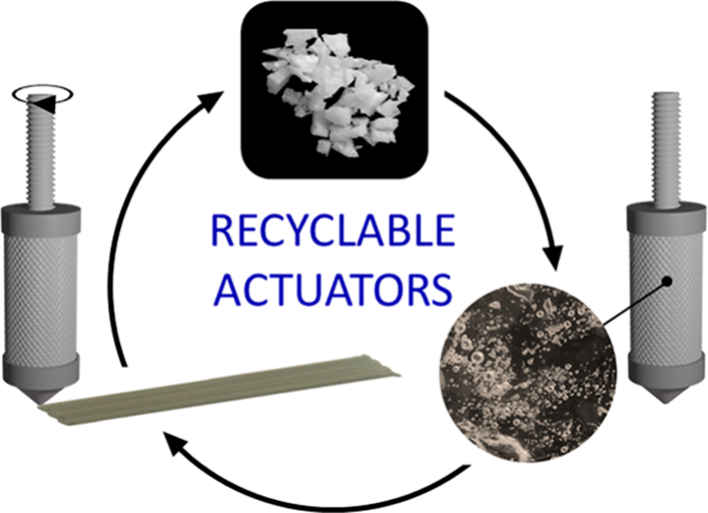

Direct ink writing (DIW) of liquid crystal elastomers (LCEs) has rapidly paved its way into the field of soft actuators and other stimuli-responsive devices. However, currently used LCE systems for DIW require postprinting (photo)polymerization, thereby forming a covalent network, making the process time-consuming and the material nonrecyclable. In this work, a DIW approach is developed for printing a supramolecular poly(thio)urethane LCE to overcome these drawbacks of permanent cross-linking. The thermo-reversible nature of the supramolecular cross-links enables the interplay between melt-processable behavior required for extrusion and formation of the network to fix the alignment. After printing, the actuators demonstrated a reversible contraction of 12.7% or bending and curling motions when printed on a passive substrate. The thermoplastic ink enables recyclability, as shown by cutting and printing the actuators five times. However, the actuation performance diminishes. This work highlights the potential of supramolecular LCE inks for DIW soft circular actuators and other devices.

Direct ink writing (DIW) of stimuli-responsive materials is a promising microextrusion technique to create complex shapes with high resolutions,^1^ which is often applied in, e.g., soft robotics,^2−5^ haptic devices,^6,7^ and structurally colored designs.^8−11^ Materials applicable as inks for DIW are hydrogels,^12,13^ shape-memory polymers,^14,15^ and liquid crystals (LCs).^16−19^ Among them, liquid crystal elastomers (LCEs) are attractive inks due to their large, reversible deformations and viscoelastic, non-Newtonian flow behavior.^20−22^ The extrusion-induced shear and elongational forces result in uniaxial molecular order along the printing path direction. Generally, photoinduced cross-linking of the printed LCEs is needed as these cross-linked polymers typically show reversible shape changes around the isotropization temperature (*T*_i_).^23−27^

To date, materials for printing soft actuators with DIW have been reported to show shape deformations in response to a variety of stimuli.^28−32^ Although the current LCE inks are well-suited for DIW to print stimuli-responsive actuators, the photopolymerization step is rather time-consuming, and efficient curing is challenging. Furthermore, the permanent cross-links hinder the material’s recyclability and the fabrication of sustainable soft actuators. In previous work, the combination of dynamic covalent^33−38^ and supramolecular cross-links has allowed for fabricating photoswitchable actuators by DIW without the need for photocuring.^39^ While highlighting the programmable shape-switching behavior, the recyclability of this printable material was not demonstrated.

An alternative way to overcome the limitations inherent to curing and permanent cross-linking is by only introducing supramolecular interactions as dynamic physical cross-links.^40−42^ Recently, we reported supramolecular soft actuators based on melt-processable poly(thio)urethane (PTU) LCEs.^43^ The segmented copolymer contains LC soft and hydrogen-bonding thiourethane (TU) hard segments. At elevated temperatures, the hydrogen bonds dissociate, allowing for melt-processable properties, whereas subsequent cooling recovers the hydrogen bonds and stabilizes the material along with its possible orientation. We now report on the use of these PTU LCEs as ink for the DIW of free-standing actuators and four recycles, showing reversible contractions up to 12.7%.

The melt-processable supramolecular LCE is synthesized through sequential thiol–acrylate Michael addition and thiol–isocyanate reactions with commonly used diacrylate mesogens for LCEs (**1** and **2**, Figure 1a), dithiols, and diisocyanates (Figure S1).^43^ An equimolar ratio of two different mesogenic moieties is used to suppress the smectic mesophase formation. The alternating responsive mesogenic and dynamic hydrogen-bonding segments consist of five and one repeating unit(s), respectively. Molecular structures and molar ratios used for all compounds can be found in Table S1 (Supporting Information). Polymerization was confirmed through Fourier-transform infrared spectroscopy (FTIR) by the disappearance of the characteristic peaks for the thiol (*ṽ* ≈ 2560 cm^–1^) and isocyanate (*ṽ* ≈ 2270 cm^–1^) stretching bands (Figure S2). Furthermore, the characteristic peaks for the hydrogen-bonded amine (*ṽ* ≈ 3315 cm^–1^) and carbonyl (*ṽ* ≈ 1638 cm^–1^) indicate the formation of TU moieties. According to gel permeation chromatography (GPC), the final reaction product has a number-average molar mass *M*_n_ of ≈ 73 kg mol^–1^ and relatively low polydispersity *Đ* = 1.90, indicating the successful synthesis of the PTUs (Figure S3).

**Figure 1:**
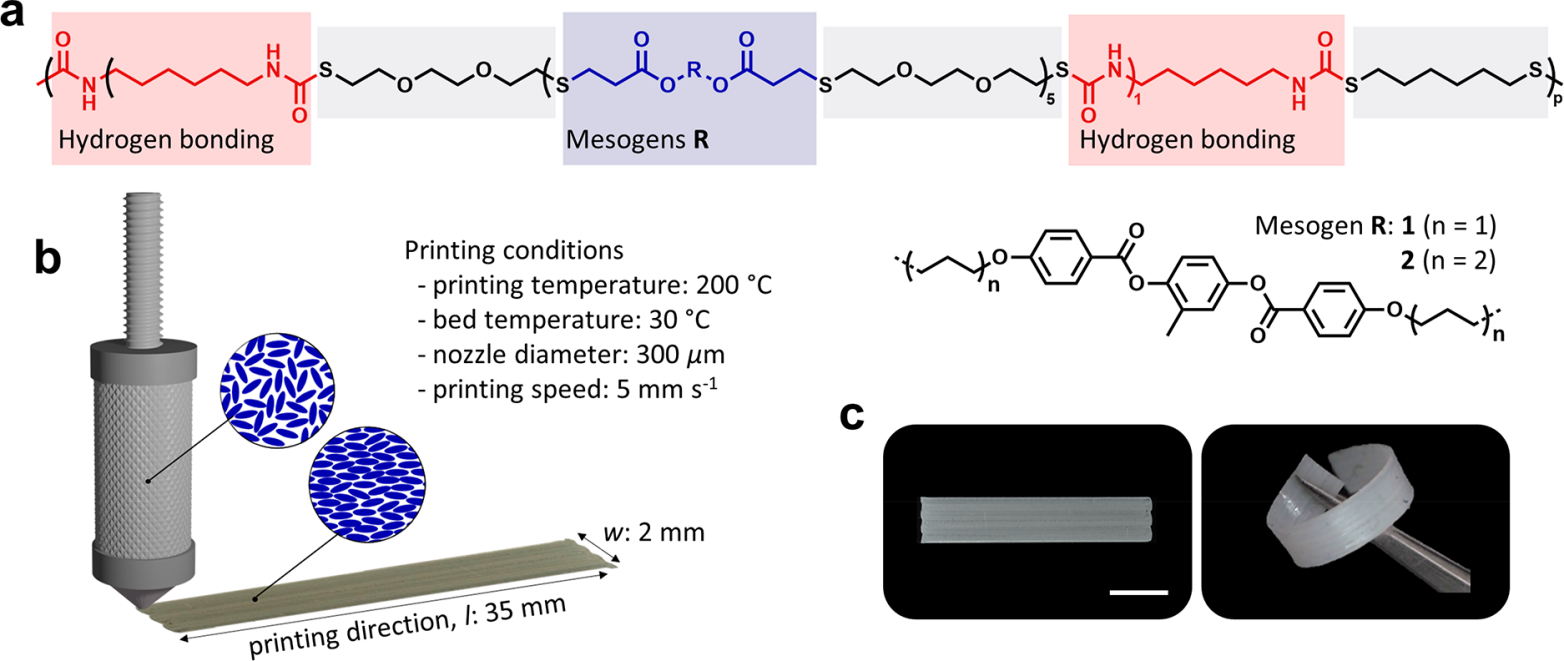
(a) Molecular representation of the segmented PTU consisting of responsive mesogens (blue) **1** and **2**, dynamic hydrogen-bonding moieties (red), and chain extenders (gray). (b) Scheme of the printing process and used printing parameters. The inset schematically shows the mesogens at 200 °C in the isotropic state and the molecular order after printing in a high shear environment. (c) Free-standing thermoplastic LCE displaying the flexibility and rubbery properties after printing. The scale bar represents 0.25 cm.

Further characterization of the PTU LCEs with differential scanning calorimetry (DSC), thermogravimetric analysis (TGA), and dynamic mechanical analysis (DMA) shows the thermal material properties that are important for DIW. The melting and degradation temperatures are observed from DSC and TGA characterizations, providing insight into the material processability range. The DSC thermogram shows an endothermic melting peak of the TU segment domains at 173 °C (Figure S4), corresponding to the material’s melting temperature and thus the minimal printing temperature for DIW, whereas from TGA, a 1% weight loss is observed around 240 °C (Figure S5) due to polymer decomposition,^44^ setting the upper limit of the processing temperature of the PTU LCE. These combined results from DSC and TGA lead to a DIW processing temperature range between around 170 and 240 °C. DMA analysis revealed the storage modulus (*E*′) inflection point and loss tangent (tan δ) peak maximum at around 4 °C, corresponding to the glass-transition temperature (Figure S6). The rubbery plateau is between 65 and 130 °C, which indicates the presence of the physical, hydrogen-bonded cross-links (Figure S7). Within this temperature range, a distinct drop in *E*′ is observed at 83 °C, indicative of the *T*_i_ of the material.^45,46^ The *T*_i_ is significantly lower than the processing temperature, which is beneficial to achieving stable actuators showing large, reversible deformations.^43^ Above 130 °C, the material enters the viscoelastic flow region, where the hydrogen bonds dissociate. This bond dissociation allows for the thermoplastic melt behavior required for extrusion in the DIW process. At the same time, cooling of the material ensures the stability of the supramolecular network through the formation of hydrogen bonds, therefore locking in the printing-induced molecular alignment after extrusion. Microphase-separated morphologies were observed with medium-angle X-ray scattering (MAXS), indicating the formation of distinct responsive LC and hydrogen-bonding TU domains (Figure S8).^43^

Based on the thermal characterization results, the processing temperatures of the syringe (*T*_syringe_) and bed (*T*_bed_) were set. Typically, DIW of LC-based inks is done around the material’s *T*_i_.^8,20^ However, in our case, much higher temperatures are needed since the melting onset is around 170 °C. Therefore, the ink is printed with *T*_syringe_ = 200 °C, at which the PTU LCE is in its thermoplastic melt and there is strong control over the extrusion. At such high temperatures (*T*_syringe_ > *T*_i_), the molecular order as well as the hydrogen-bonding cross-links are highly reduced. The thermal gradient from the heated ink reservoir to the substrate results in immediate locking of the extrusion-induced uniaxial alignment after printing due to the thermo-reversible character of the hydrogen bonds. As the deposited material continues to cool on the printing bed, the LC-ordered state is formed. For this reason, and to maintain this parameter constant, *T*_bed_ was set at 30 °C. Furthermore, a tapered nozzle of 300 μm in diameter and a lateral nozzle speed of 5 mm s^–1^ were used. DIW-printed PTU LCE actuators are fabricated following these printing settings and a programmed print path (Figure 1b; Table S2 and Figure S9, Supporting Information). Video S1 (Supporting Information) shows the printing process of uniaxially oriented soft actuators. Depositing a single layer (35 × 2 mm^2^) on top of a polyvinylpyrrolidone (PVP) coated glass substrate and subsequently dissolving this sacrificial PVP layer yield free-standing actuator films (Figure 1c). Further characterization takes place on films with dimensions of 25 × 2 mm^2^, and the obtained thickness by an optical profiling system is averaged to 140 μm (Figure S10).

Wide-angle X-ray scattering (WAXS) of the printed free-standing actuator verifies the DIW-induced uniaxial molecular order. The resulting 2D WAXS diffractogram of the pristine material actuator shows typical orientationally arranged diffraction patterns orthogonal to the printing direction, indicating uniaxial alignment with an order parameter *S* = 0.16 (Figures S11 and S12). After confirming the presence of molecular order, thermal actuation between 30 and 110 °C demonstrates the thermal-responsive behavior (Figure 2a). From the thermal-induced contraction along the long axis, the actuation strain is calculated as ε_a_ = −(*l – l*_0_)/*l*_0_, where *l*_0_ is the length of the LCE film at room temperature and *l* is the length at a specific temperature. The resulting thermal-actuation response displays an immediate contraction upon heating to 110 °C with a maximum actuation strain ε_a_ = 12.7% (Figure 2b; Figure S13). In addition, the actuation behavior of different printed pristine actuators shows similar performance (Figure S13). Comparing the actuators obtained by DIW with our previously reported mechanically programmed actuators (ε_a_ = 32%),^43^ less contraction is observed for the DIW-printed actuator upon heating, which also correlates to the lower order parameter. Our results reveal that uniaxially aligned thermoplastic PTU LCE actuators were successfully obtained by DIW without the need for photocuring, showing reversible actuation over at least three heating–cooling cycles (Figure 2c).

**Figure 2:**
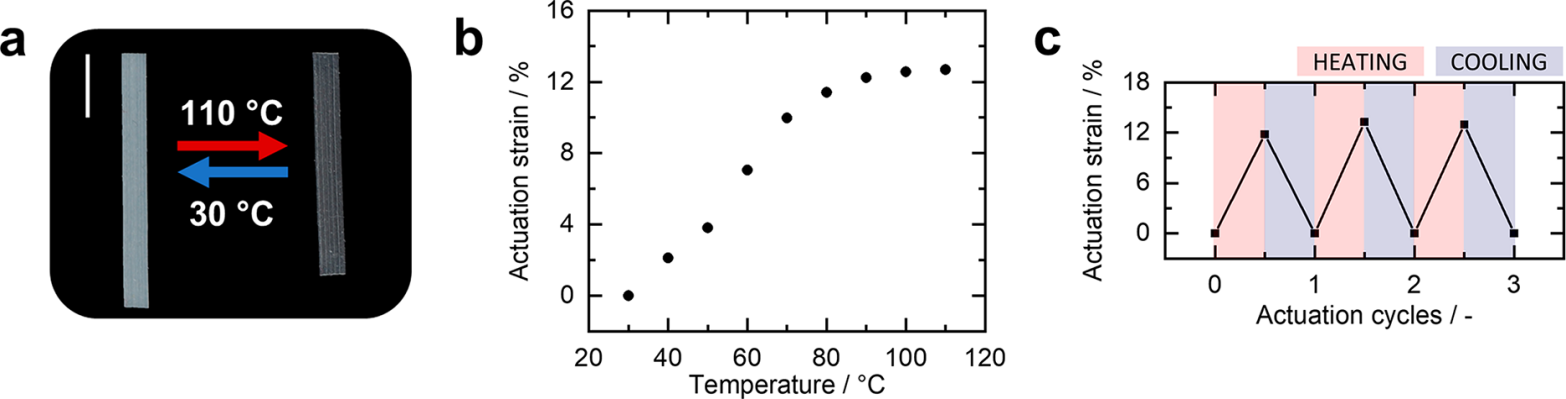
(a) Temperature response of the printed PTU LCE actuator by heating it from 30 to 110 °C. The scale bar represents 0.50 cm. (b) The corresponding actuation strain as a function of temperature showing a maximum contraction up to 12.7%. The actuation performance is averaged over three heating–cooling cycles. (c) Thermal cycling of the printed strip by repeated heating and cooling between 110 and 30 °C.

Multiple cycles of the thermoplastic PTU LCE actuators were printed, exploiting their dynamic nature and showing the materials’ circularity. Figure 3a displays the recycling procedure: first, the thermoplastic material is cut into small pieces and loaded into the syringe, where it is heated to *T*_syringe_ = 200 °C again for 20 min to ensure complete melting. Extrusion of the thermoplastic polymer melt with the set print parameters yields the actuators. When printed and cooled to room temperature, the material is recut again into small pieces and reloaded into the syringe without further treatments to recycle the material. The same batch of the thermoplastic material was printed five times through DIW, yielding actuators from the initial pristine material and an additional four recycles. The pristine material actuator contracts with a maximum of 12.7%, while the first and second recycles have a comparable actuation strain of 9.9% and 10.0%, respectively. The third- and fourth-times recycled actuators show diminished actuation strains of 3.3% and 0.7%, respectively (Figures 3b and 3c). To shed light on this diminished actuation performance, X-ray diffraction, TGA, and GPC analysis were performed.

**Figure 3:**
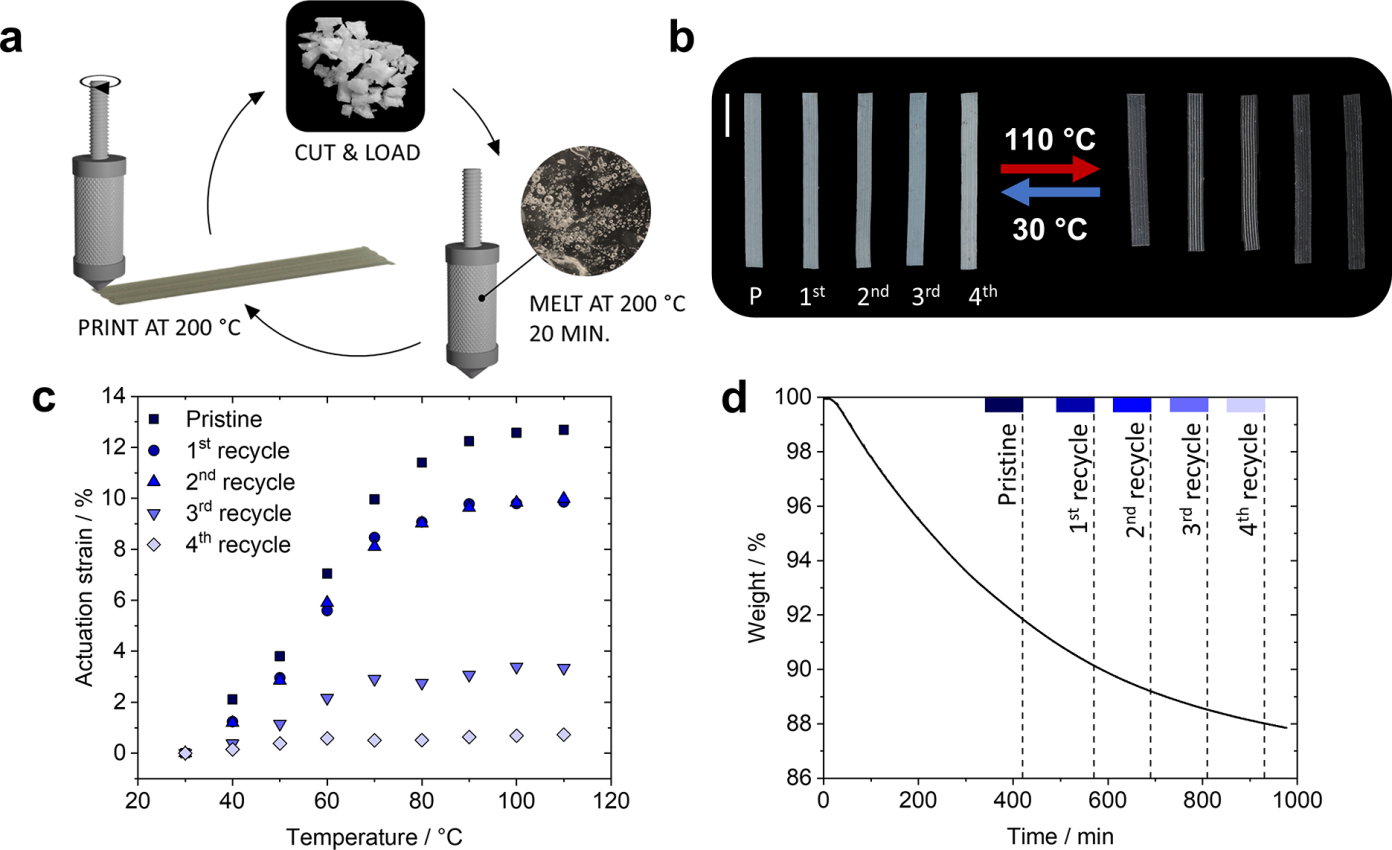
(a) Scheme of recycling procedure wherein the LCE is printed for five cycles. First, the crude material is cut and heated in the syringe to 200 °C for 20 min, after which pristine uniaxial actuators are printed. The extruded material is then recut, and the procedure is repeated to print actuators with the recycled material. (b) Photographs of the pristine (P) to fourth recycle actuators (from left to right) at 30 and 110 °C. The scale bar represents 0.50 cm. (c) The corresponding actuation strain of the printed actuators as a function of the temperature. The actuation performance of each printed actuator is averaged over three heating–cooling cycles. (d) TGA data showing the weight loss of the PTU when heated to 200 °C for 16 h. The dashed lines indicate the melting time of each printing cycle.

The orientationally arranged diffraction patterns in the wide-angle 2D X-ray diffractograms and the accompanying order parameter indicate the presence of extrusion-induced order in all printing cycles (Figures S11 and S12). However, the molecular alignment diminishes with ascending printing (re)cycles. The first recycle has an *S* of 0.18, slightly larger than the initial pristine cycle (*S* = 0.16), whereas the second recycle has a similar *S* of 0.15. After this, the molecular order decreases significantly to *S* = 0.09 for the third and to *S* = 0.03 for the fourth recycle. The stability of the LCE ink was evaluated with GPC and TGA. From GPC, a decreasing number-average molar mass is observed upon printing and recycling from *M*_n_ = 73 to 21 kg mol^–1^ (Figure S14 and Table S3). Heating the crude PTU LCE material to 200 °C results in a weight loss of 12 wt % within 16 h, indicating the thermal decomposition of the material (Figure 3d). This behavior might be due to the decomposition of TU moieties,^44^ which corresponds to around 10 wt % of the material and is responsible for physical cross-linking. Our conjecture is confirmed by analyzing the thermal properties with DSC, revealing that the melting endotherm of the TU hard segment domains is diminished and decreased to 137 °C upon recycling (Figure S4).

These combined observations of obtaining shorter polymer chain lengths and significant weight loss indicate the thermal decomposition of the printed material when prolonged to heat, which results in a lower extrusion-induced order during printing and is accompanied by a decrease in actuation performance. Therefore, the decrease in order parameter and actuation performance can be explained by thermal degradation of the material during printing, given that it is heated to 200 °C for 16 h in order to print all cycles.

Our ink can also be used to fabricate bending actuators by printing on a passive substrate. Depositing a PTU LCE stripe (25 × 2 mm^2^) on top of a PEI foil (25 × 5 × 0.005 mm^3^, 40% coverage) enables bending motions upon heating with the LCE on the inside of the curvature (Figure 4a). When fixing the printed film on one side, it initially bends and then tightly rolls up (Figure S15). This behavior results from the contraction of the LCE along its alignment direction, whereas the passive layer is unresponsive, resulting in bending and curling motions.^16^ Further, selective bending of more complex substrate shapes can be obtained by applying the active LCE material (35 × 2 mm^2^) to specific regions (Figure 4b).

**Figure 4:**
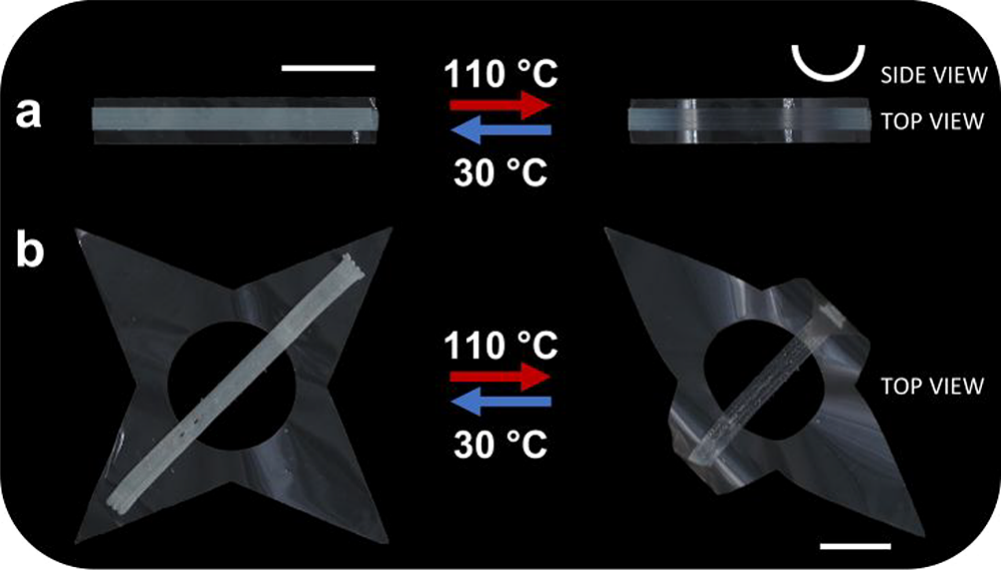
Photographs demonstrating the thermal actuation of patterned actuators by applying an LCE stripe (25–35 × 2 mm^2^) on top of (a) rectangular and (b) star-shaped passive PEI films (top view). The white stripe denotes the flat state, while the arc represents the bent state (side view). The scale bar represents 0.50 cm (bottom right).

In conclusion, we have fabricated supramolecular PTU LCE actuators through DIW without the need for postprinting (photo)polymerization, showing a maximum actuation strain of 12.7%. Due to the TU segments’ thermoreversible hydrogen bonding, the thermoplastic ink exploits the interplay between melt-processable material behavior required for extrusion and provides the desired mechanical stability to fix the network and its molecular order. Furthermore, the ink can be recycled, but the actuation performance diminishes due to the thermal degradation of the thermoplastic LCE ink. We foresee that the thermal degradation can be circumvented by adapting the chemical structure, changing the printing conditions, and/or the use of fused filaments. This supramolecular, thermoplastic ink demonstrates an innovative approach toward the DIW of recyclable LCE actuators where photocuring is not needed, which opens up great opportunities for the facile fabrication of more sustainable soft actuators and robotic devices. Currently, we are exploiting the DIW of more complex 3D-shaped actuators using our supramolecular ink.

## Supporting Information Available

Printing process of uniaxially aligned thermoplastic LCE strips (MP4)
Experimental details on synthesis, characterizations, direct ink writing, and additional results (PDF)

## References

[ref1] Ligon S. C.; Liska R.; Stampfl J.; Gurr M.; Mülhaupt R. Polymers for 3D Printing and Customized Additive Manufacturing. Chem. Rev. 2017, 117 (15), 10212–10290. 10.1021/acs.chemrev.7b00074. 28756658 PMC5553103

[ref2] López-Valdeolivas M.; Liu D.; Broer D. J.; Sánchez-Somolinos C. 4D Printed Actuators with Soft-Robotic Functions. Macromol. Rapid Commun. 2018, 39 (5), 1700710 10.1002/marc.201700710. 29210486

[ref3] Kotikian A.; McMahan C.; Davidson E. C.; Muhammad J. M.; Weeks R. D.; Daraio C.; Lewis J. A. Untethered Soft Robotic Matter with Passive Control of Shape Morphing and Propulsion. Sci. Robot. 2019, 4 (33), eaax7044 10.1126/scirobotics.aax7044. 33137783

[ref4] McCracken J. M.; Donovan B. R.; White T. J. Materials as Machines. Adv. Mater. 2020, 32 (20), 1906564 10.1002/adma.201906564. 32133704

[ref5] Huang S.; Huang Y.; Li Q. Photodeformable Liquid Crystalline Polymers Containing Functional Additives: Toward Photomanipulatable Intelligent Soft Systems. Small Struct 2021, 2 (9), 2100038 10.1002/sstr.202100038.

[ref6] Zhai Y.; Wang Z.; Kwon K. S.; Cai S.; Lipomi D. J.; Ng T. N. Printing Multi-Material Organic Haptic Actuators. Adv. Mater. 2021, 33 (19), 2002541 10.1002/adma.202002541. 33135205

[ref7] Li S.; Bai H.; Shepherd R. F.; Zhao H. Bio-Inspired Design and Additive Manufacturing of Soft Materials, Machines, Robots, and Haptic Interfaces. Angew. Chem. Int. Ed 2019, 58 (33), 11182–11204. 10.1002/anie.201813402. 30707785

[ref8] Sol J. A. H. P.; Smits L. G.; Schenning A. P. H. J.; Debije M. G. Direct Ink Writing of 4D Structural Colors. Adv. Funct. Mater. 2022, 2201766 10.1002/adfm.202201766.

[ref9] Kim J. B.; Chae C.; Han S. H.; Lee S. Y.; Kim S. H. Direct Writing of Customized Structural-Color Graphics with Colloidal Photonic Inks. Sci. Adv. 2021, 7 (48), eabj8780 10.1126/sciadv.abj8780. 34818030 PMC8612532

[ref10] Chan C. L. C.; Lei I. M.; van de Kerkhof G. T.; Parker R. M.; Richards K. D.; Evans R. C.; Huang Y. Y. S.; Vignolini S. 3D Printing of Liquid Crystalline Hydroxypropyl Cellulose—toward Tunable and Sustainable Volumetric Photonic Structures. Adv. Funct. Mater. 2022, 32 (15), 2108566 10.1002/adfm.202108566.

[ref11] Wang Z.; Guo Y.; Cai S.; Yang J. Three-Dimensional Printing of Liquid Crystal Elastomers and Their Applications. ACS Appl. Polym. Mater. 2022, 4 (5), 3153–3168. 10.1021/acsapm.1c01598.

[ref12] Karis D. G.; Ono R. J.; Zhang M.; Vora A.; Storti D.; Ganter M. A.; Nelson A. Cross-Linkable Multi-Stimuli Responsive Hydrogel Inks for Direct-Write 3D Printing. Polym. Chem. 2017, 8 (29), 4199–4206. 10.1039/C7PY00831G.

[ref13] Cheng Y.; Chan K. H.; Wang X. Q.; Ding T.; Li T.; Lu X.; Ho G. W. Direct-Ink-Write 3D Printing of Hydrogels into Biomimetic Soft Robots. ACS Nano 2019, 13 (11), 13176–13184. 10.1021/acsnano.9b06144. 31625724

[ref14] Guo Y.; Liu Y.; Liu J.; Zhao J.; Zhang H.; Zhang Z. Shape Memory Epoxy Composites with High Mechanical Performance Manufactured by Multi-Material Direct Ink Writing. Compos. Part A Appl. Sci. Manuf. 2020, 135, 105903 10.1016/j.compositesa.2020.105903.

[ref15] Zhang Y.; Yin X. Y.; Zheng M.; Moorlag C.; Yang J.; Wang Z. L. 3D Printing of Thermoreversible Polyurethanes with Targeted Shape Memory and Precise in Situ Self-Healing Properties. J. Mater. Chem. A 2019, 7 (12), 6972–6984. 10.1039/C8TA12428K.

[ref16] Pozo M. d.; Sol J. A. H. P.; van Uden S. H. P.; Peeketi A. R.; Lugger S. J. D.; Annabattula R. K.; Schenning A. P. H. J.; Debije M. G. Patterned Actuators via Direct Ink Writing of Liquid Crystals. ACS Appl. Mater. Interfaces 2021, 13 (49), 59381–59391. 10.1021/acsami.1c20348. 34870984 PMC8678986

[ref17] Narupai B.; Nelson A. 100th Anniversary of Macromolecular Science Viewpoint: Macromolecular Materials for Additive Manufacturing. ACS Macro Lett. 2020, 9 (5), 627–638. 10.1021/acsmacrolett.0c00200. 35648567

[ref18] Kuang X.; Roach D. J.; Wu J.; Hamel C. M.; Ding Z.; Wang T.; Dunn M. L.; Qi H. J. Advances in 4D Printing: Materials and Applications. Adv. Funct. Mater. 2019, 29 (2), 1805290 10.1002/adfm.201805290.

[ref19] Bisoyi H. K.; Li Q. Liquid Crystals: Versatile Self-Organized Smart Soft Materials. Chem. Rev. 2022, 122 (5), 4887–4926. 10.1021/acs.chemrev.1c00761. 34941251

[ref20] del Pozo M.; Sol J. A. H. P.; Schenning A. P. H. J.; Debije M. G. 4D Printing of Liquid Crystals: What’s Right for Me?. Adv. Mater. 2022, 34 (3), 2104390 10.1002/adma.202104390. 34716625

[ref21] Bauman G. E.; Mccracken J. M.; White T. J. Actuation of Liquid Crystalline Elastomers at or Below Ambient Temperature. Angew. Chem. Int. Ed 2022, e202202577. 10.1002/anie.202202577 35482590

[ref22] Zhang C.; Lu X.; Fei G.; Wang Z.; Xia H.; Zhao Y. 4D Printing of a Liquid Crystal Elastomer with a Controllable Orientation Gradient. ACS Appl. Mater. Interfaces 2019, 11 (47), 44774–44782. 10.1021/acsami.9b18037. 31692319

[ref23] Ren L.; Li B.; He Y.; Song Z.; Zhou X.; Liu Q.; Ren L. Programming Shape-Morphing Behavior of Liquid Crystal Elastomers via Parameter-Encoded 4D Printing. ACS Appl. Mater. Interfaces 2020, 12 (13), 15562–15572. 10.1021/acsami.0c00027. 32157863

[ref24] Kotikian A.; Truby R. L.; Boley J. W.; White T. J.; Lewis J. A. 3D Printing of Liquid Crystal Elastomeric Actuators with Spatially Programed Nematic Order. Adv. Mater. 2018, 30 (10), 1706164 10.1002/adma.201706164. 29334165

[ref25] Wang Z.; Wang Z.; Zheng Y.; He Q.; Wang Y.; Cai S. Three-Dimensional Printing of Functionally Graded Liquid Crystal Elastomer. Sci. Adv. 2020, 6 (39), eabc0034 10.1126/sciadv.abc0034. 32978149 PMC7518867

[ref26] Saed M. O.; Ambulo C. P.; Kim H.; De R.; Raval V.; Searles K.; Siddiqui D. A.; Cue J. M. O.; Stefan M. C.; Shankar M. R.; Ware T. H. Molecularly-Engineered, 4D-Printed Liquid Crystal Elastomer Actuators. Adv. Funct. Mater. 2019, 29 (3), 1806412 10.1002/adfm.201806412.

[ref27] Liu Z.; Bisoyi H. K.; Huang Y.; Wang M.; Yang H.; Li Q. Thermo- and Mechanochromic Camouflage and Self-Healing in Biomimetic Soft Actuators Based on Liquid Crystal Elastomers. Angew. Chem. Int. Ed 2022, 61 (8), e202115755. 10.1002/anie.202115755 34904346

[ref28] Davidson E. C.; Kotikian A.; Li S.; Aizenberg J.; Lewis J. A. 3D Printable and Reconfigurable Liquid Crystal Elastomers with Light-Induced Shape Memory via Dynamic Bond Exchange. Adv. Mater. 2020, 32 (1), 1905682 10.1002/adma.201905682. 31664754

[ref29] del Pozo M.; Liu L.; Pilz da Cunha M.; Broer D. J.; Schenning A. P. H. J. Direct Ink Writing of a Light-Responsive Underwater Liquid Crystal Actuator with Atypical Temperature-Dependent Shape Changes. Adv. Funct. Mater. 2020, 30 (50), 2005560 10.1002/adfm.202005560.

[ref30] Ambulo C. P.; Burroughs J. J.; Boothby J. M.; Kim H.; Shankar M. R.; Ware T. H. Four-Dimensional Printing of Liquid Crystal Elastomers. ACS Appl. Mater. Interfaces 2017, 9 (42), 37332–37339. 10.1021/acsami.7b11851. 28967260

[ref31] Kim K.; Guo Y.; Bae J.; Choi S.; Song H. Y.; Park S.; Hyun K.; Ahn S. K. 4D Printing of Hygroscopic Liquid Crystal Elastomer Actuators. Small 2021, 17 (23), 2100910 10.1002/smll.202100910. 33938152

[ref32] Ceamanos L.; Kahveci Z.; Lopez-Valdeolivas M.; Liu D.; Broer D. J.; Sanchez-Somolinos C. Four-Dimensional Printed Liquid Crystalline Elastomer Actuators with Fast Photoinduced Mechanical Response toward Light-Driven Robotic Functions. ACS Appl. Mater. Interfaces 2020, 12 (39), 44195–44204. 10.1021/acsami.0c13341. 32885661

[ref33] Li X.; Yu R.; He Y.; Zhang Y.; Yang X.; Zhao X.; Huang W. Self-Healing Polyurethane Elastomers Based on a Disulfide Bond by Digital Light Processing 3D Printing. ACS Macro Lett. 2019, 8 (11), 1511–1516. 10.1021/acsmacrolett.9b00766. 35651184

[ref34] Zheng M.; Guo Q.; Yin X.; Getangama N. N.; de Bruyn J. R.; Xiao J.; Bai Y.; Liu M.; Yang J. Direct Ink Writing of Recyclable and in Situ Repairable Photothermal Polyurethane for Sustainable 3D Printing Development. J. Mater. Chem. A 2021, 9 (11), 6981–6992. 10.1039/D0TA11341G.

[ref35] Robinson L. L.; Self J. L.; Fusi A. D.; Bates M. W.; Read De Alaniz J.; Hawker C. J.; Bates C. M.; Sample C. S. Chemical and Mechanical Tunability of 3D-Printed Dynamic Covalent Networks Based on Boronate Esters. ACS Macro Lett. 2021, 10 (7), 857–863. 10.1021/acsmacrolett.1c00257. 35549203

[ref36] Huang S.; Shen Y.; Bisoyi H. K.; Tao Y.; Liu Z.; Wang M.; Yang H.; Li Q. Covalent Adaptable Liquid Crystal Networks Enabled by Reversible Ring-Opening Cascades of Cyclic Disulfides. J. Am. Chem. Soc. 2021, 143 (32), 12543–12551. 10.1021/jacs.1c03661. 34275290

[ref37] Jiang Z.; Xiao Y.; Cheng R.; Hou J.; Zhao Y. Dynamic Liquid Crystalline Networks for Twisted Fiber and Spring Actuators Capable of Fast Light-Driven Movement with Enhanced Environment Adaptability. Chem. Mater. 2021, 33 (16), 6541–6552. 10.1021/acs.chemmater.1c02073.

[ref38] Chen L.; Bisoyi H. K.; Huang Y.; Huang S.; Wang M.; Yang H.; Li Q. Healable and Rearrangeable Networks of Liquid Crystal Elastomers Enabled by Diselenide Bonds. Angew. Chem., Int. Ed. 2021, 60 (30), 16394–16398. 10.1002/anie.202105278. 33977661

[ref39] Lu X.; Ambulo C. P.; Wang S.; Rivera-Tarazona L. K.; Kim H.; Searles K.; Ware T. H. 4D-Printing of Photoswitchable Actuators. Angew. Chem. Int. Ed 2021, 60 (10), 5536 10.1002/anie.202012618. 33217118

[ref40] Sun J.; Peng B.; Lu Y.; Zhang X.; Wei J.; Zhu C.; Yu Y. A Photoorganizable Triple Shape Memory Polymer for Deployable Devices. Small 2022, 18 (9), 2106443 10.1002/smll.202106443. 34918481

[ref41] Zhou Y.; Wang L.; Ma S.; Zhang H. Fully Room-Temperature Reprogrammable, Reprocessable, and Photomobile Soft Actuators from a High-Molecular-Weight Main-Chain Azobenzene Crystalline Poly(Ester-Amide). ACS Appl. Mater. Interfaces 2022, 14 (2), 3264–3273. 10.1021/acsami.1c18647. 34991314

[ref42] Ube T.; Nakayama R.; Ikeda T. Photoinduced Motions of Thermoplastic Polyurethanes Containing Azobenzene Moieties in Main Chains. Macromolecules 2022, 55 (2), 413–420. 10.1021/acs.macromol.1c01827.

[ref43] Lugger S. J. D.; Mulder D. J.; Schenning A. P. H. J. One-Pot Synthesis of Melt-Processable Supramolecular Soft Actuators. Angew. Chem. Int. Ed 2022, 61 (6), e202115166. 10.1002/anie.202115166 PMC9300041 34826175

[ref44] Ireni N. G.; Narayan R.; Basak P.; Raju K. V. S. N. Poly(Thiourethane-Urethane)-Urea as Anticorrosion Coatings with Impressive Optical Properties. Polymer 2016, 97, 370–379. 10.1016/j.polymer.2016.04.072.

[ref45] Hotta A.; Terentjev E. M. Dynamic Soft Elasticity in Monodomain Nematic Elastomers. Eur. Phys. J. E 2003, 10 (4), 291–301. 10.1140/epje/i2002-10005-5. 15156585

[ref46] Merkel D. R.; Traugutt N. A.; Visvanathan R.; Yakacki C. M.; Frick C. P. Thermomechanical Properties of Monodomain Nematic Main-Chain Liquid Crystal Elastomers. Soft Matter 2018, 14 (29), 6024–6036. 10.1039/C8SM01178H. 29974115

