# Supplementary material for: Direct Ink Writing of Recyclable Supramolecular Soft Actuators
Source: ACS Macro Lett. 2022 Jul 8;11(7):935–40. doi: 10.1021/acsmacrolett.2c00359 (PMC9301911; doi:10.1021/acsmacrolett.2c00359)
Supplement: Supplementary file 2 — mz2c00359_si_002.pdf [file mz2c00359_si_002.pdf]

SUPPORTING INFORMATION

Direct Ink Writing of Recyclable Supramolecular Soft Actuators

Sean J. D. Lugger,[1] Ruth M. C. Verbroekken,[1] Dirk J. Mulder,[1] and Albert P. H. J. Schenning[1,2,*]

[1]Laboratory of Stimuli-Responsive Functional Materials and Devices (SFD), Department of Chemical Engineering and Chemistry, Eindhoven University of Technology (TU/e), P.O. Box 513, 5600 MB Eindhoven, The Netherlands. E-mail: a.p.h.j.schenning@tue.nl.

[2]Institute for Complex Molecular Systems, Eindhoven University of Technology (TU/e), P.O. Box 513, 5600 MB Eindhoven, The Netherlands.

Table of Contents

Experimental Procedures 1

Supporting Tables and Figures 3

Supporting Video Caption 9

References 9

Experimental Procedures

Materials

1,4-Bis-[4-(3-acryloyloxypropyloxy)benzoyloxy]-2-methylbenzene (**1**) and 1,4-Bis-[4-(6-acryloyloxyhexyloxy)benzoyloxy]-2-methylbenzene (**2**) were obtained from Merck. 2,2′-(Ethylenedioxy)diethanethiol (**3**, ≥97%), Hexamethylene diisocyanate (**5**, ≥98%), Triethylamine (**6**, ≥99%), and 1,6-Hexanedithiol (**7**, ≥97%) were purchased from Tokyo Chemical Industry (TCI). Dimethylphenylphosphine (**4**, 99%) and *N*,*N*-Dimethylacetamide (DMAc, ≥99%) were purchased from Sigma-Aldrich. Diethyl ether (Et_2O, ≥99.5%) was obtained from Biosolve. To prepare the sacrificial layer, polyvinylpyrrolidone (PVP; M_w 10,000) was purchased from Sigma-Aldrich. All reagents were used as received without further purification.

Synthetic procedure

The supramolecular cross-linked thermoplastic LCE is obtained by sequential thiol-acrylate and thiol-isocyanate addition reactions (Figure S1, Table S1). In short, reactive mesogens **1** (15.84 mmol, 9.312 g) and **2** (15.84 mmol, 10.6597 g) were added in a 1:1 molar ratio to a 250 mL round-bottom vessel, after which they were dissolved in DMAc 50 wt% (42 mL) at 50 °C under Ar atmosphere. Next, the temperature is brought back to room temperature, and an excess of **3** (38.00 mmol, 6.9394 g) and 0.1 wt% of **4** (0.28 mmol, 0.0381 g) are added. After allowing the reaction to proceed for two hours, the prepolymer is sequentially prepared via a base-catalyzed thiol-isocyanate addition reaction. For this reaction, an aliphatic diisocyanate **5** (12.70 mmol, 2.1330 g) in DMAc 50 wt% (2 mL) and an amine catalyst **6** (0.1 wt%, 0.34 mmol, 0.0346 g) is added to the thiol-terminated oligomer. After 15 minutes at room temperature, the viscosity increased, and therefore, additional DMAc (40 mL) was added to the mixture. Next, a 1:1 molar ratio of the dithiol chain extender **7** (6.34 mmol, 0.9533 g) is added to the oligomer prepolymer, after which the reaction proceeds overnight at 60 °C. The final polymer is obtained through precipitation in cold Et_2O (2.5 L) overnight and subsequently stirred in fresh cold Et_2O (400 mL) for additional 3.5 hours, yielding the PTU LCE (96.2% recovery). Finally, the material was dried at 40 °C under vacuum overnight.

Characterization

FTIR spectra were recorded on a Varian 670 IR spectrometer equipped with an attenuated total reflectance (ATR) sampling accessory using a diamond crystal from 4000 to 650 cm^{-1}, collecting 50 scans per spectrum with a spectral resolution of 4 cm^{-1}. The FTIR spectra are recorded at room temperature and are processed with Varian Resolutions. Gel permeation chromatography (GPC) was performed on a Water HPLC system equipped with a PSS PFG (8 × 50 mm^2, 7 μm) and two PFG linear XL columns (8 × 300 mm^2, 7 μm) in series. The mobile phase, 1,1,1,3,3,3-hexafluoro-2-propanol (HFIP) with potassium trifluoroacetate (20 mM) at 35 °C, is supplied at a flow rate of 0.8 mL min^{-1}. The samples were prepared at room temperature in HFIP with potassium trifluoroacetate (20 mM) and toluene (20 mM). The molecular weights were determined using a refractive index detector relative to poly(methyl methacrylate) standards. Differential scanning calorimetry (DSC) thermographs are measured with a TA Instruments Q1000 DSC instrument, using hermetic T-zero aluminum sample pans. All scans were conducted with 10 ± 1 mg polymer over the temperature range from -50 to 210 °C with heating and cooling rates of 10 °C min^{-1} under N_2 atmosphere. The transition temperatures are determined from the second heating cycle. TGA was carried out on a TA instruments Q50 instrument with 5.1 mg polymer over a temperature range from 28 to 800 °C and a heating rate of 10 °C min^{-1}. For the thermal degradation test with TGA, 7.6 mg polymer was heated to 200 °C for 16 hours. Dynamic mechanical analysis (DMA) was performed with a TA Instruments Q800 apparatus in vertical tension mode on samples of (8.8 × 5.7 × 0.4 mm^3) cut from compression-molded films. The thermographs were measured between -50 and 250 °C, with a heating rate of 5 °C min^{-1}, a single 1 Hz oscillating frequency, 20 μm amplitude, and 0.01 N preload force. X-ray scattering measurements were performed on a Ganesha lab instrument equipped with a Genix-Cu ultralow divergence source. This source generates X-ray photons with a wavelength and flux of 0.154 nm and 1 × 10^8 photons s^{-1}, respectively. Diffraction patterns were obtained using a Pilatus 300 K silicon pixel detector with 487 × 619 pixels of 172 × 172 μm^2. As a calibration standard, silver behenate was used. The sample-to-detector distance is 89 mm for wide-angle (WAXS) configurations, whereas for medium angle (MAXS) configurations a distance of 440 mm is used. After data collection, a custom Python script with the PyFAI software package

is used to reduce and analyze the obtained data. The orientational order parameters were calculated from the diffraction patterns using the Kratky method.[1]

Preparation of Glass Printing Substrates

Free-standing actuators were fabricated by printing on glass substrates (76 × 52 mm^2, Paul Marienfeld GmbH & Co. KG) prepared with a sacrificial layer. First, the glass was cleaned by ultrasonication in 1:1 vol% 2-propanol:ethanol for 20 min (Branson 5510), after which the residual solvent was removed with N_2 gas flushing. Next, a 10 wt% polyvinylpyrrolidone (PVP) in ethanol was cast on a glass substrate during spin coating (Karl Süss RC8). The spin coater was set to 3000 rpm, 300 rpm s^{-1} acceleration, and 45 s spin time. After spin coating, the treated substrates were post-treated at 100°C for 10 min.

Direct Ink Writing

A Hyrel EHR 3D printer equipped with a TAM-15 high-operating temperature syringe extrusion head was used for the controlled deposition of the thermoplastic PTU LCE. For this, a stainless-steel ink syringe and a 0.3 mm diameter micronozzle (Micro-Swiss MK8 M2549-03 3D) were used. The printing procedure is started by cutting the PTU LCEs into small chunks and drying them at 40 °C under a vacuum overnight. Subsequently, the syringe is loaded with the dried material and heated up to 200 °C for 20 min to ensure complete melting. Extrusion of the thermoplastic melt with the found settings induces molecular order parallel to the printing direction. The remaining material inside the syringe is emptied into a PTFE dish following the same print settings, mimicking the actuator formation while minimizing the exposure to PVP. For the recycling procedure, pristine printed actuators are cut into small pieces and reloaded into the syringe without further treatments to recycle the pristine printed material. Finally, the printing procedure is repeated as described previously to fabricate recycled actuators (1st – 4th recycle). It should be noted that the crude material was heated for seven hours at 200 °C to yield the initial pristine actuators, and additional two hours of heating was required for each printing recycle, resulting in prolonged heat exposure of 15.5 hours after printing the complete recycling set. The actuation properties, thermal degradation, and alignment of the resulting prints were characterized using ImageJ image processing techniques, GPC, and X-ray scattering measurements, respectively. All print path files were obtained by manually writing the .g-code according to the desired printing conditions (speed, layer height, line spacing, and infill settings). With this technique, free-standing and patterned actuators were printed.

Patterned actuators

Patterned actuators are created by printing on 5 and 10 μm uniaxially stretched polyetherimide (PEI) foils with the reported settings. The PEI films are passive substrates that do not act as an alignment layer for oligomeric mesogens.[2] Two different pattern actuators are created with the produced .gcode files. The first is a 2 × 35 mm^2 ($w \times l$) PTU LCE film deposited on top of a 5 × 35 mm^2 ($w \times l$) PEI film. After deposition of this film, laser cutting was employed to extract a 5 × 25 mm^2 ($w \times l$) film (40% coverage).[2] In addition, a 2 × 35 mm^2 ($w \times l$) LCE film is deposited on a star-shaped PEI foil. This PEI foil is laser-cut according to a manually written vector path.

Laser Cutting

Precise cutting of the LCE patterned PEI foils was performed using a Laser Engraving Machine (CO_2 Laser Plotter 50W 40 × 40, XM-3040), equipped with a 50 W max. CO_2 laser beam. The cutting path used to slice the samples was defined using the RDWorksV8 V8.01.18 software with minimal (7.5%) and maximal (8.3%) laser intensities and cutting speed (300 mm s^{-1}) set. After creating the desired cutting path, the file can be saved into a .rd (R documentation) file that can be loaded into the laser engraving machine.

Optical Profiling

The layer thickness of the multilayer films was measured with an optical profiling system (Sensofar S neox 3D Optical profiler, equipped with a 10× objective). With the profiling system, 3D profiles were recorded over the sample width of four consecutive samples yielding the average printed thicknesses.

Actuation performance

The thermal actuation was performed by placing the printed samples on a black aluminum sheet on a hotplate. The hotplate is heated from 30 to 110 °C with 10 °C temperature intervals. After reaching 110 °C, the samples were allowed to cool back to room temperature by removal from the hotplate. One full heating and cooling cycle was performed before the actuation measurements to eliminate any possible thermal history of all prints. Subsequently, three heating-cooling cycles of each printed actuator were averaged to obtain the reported actuation strain. Photographs of the samples are made with an Olympus OM-D E-M10 Mark III (with M.Zuiko 60 mm *f*/2.8 macro lens) in manual mode, and the samples were exposed to a LED light source for proper lighting. The actuation of all free-standing actuators is calculated on these photographs via ImageJ calculations. For this, the measurement of the reference sample sets the scale.

Supporting Tables and Figures

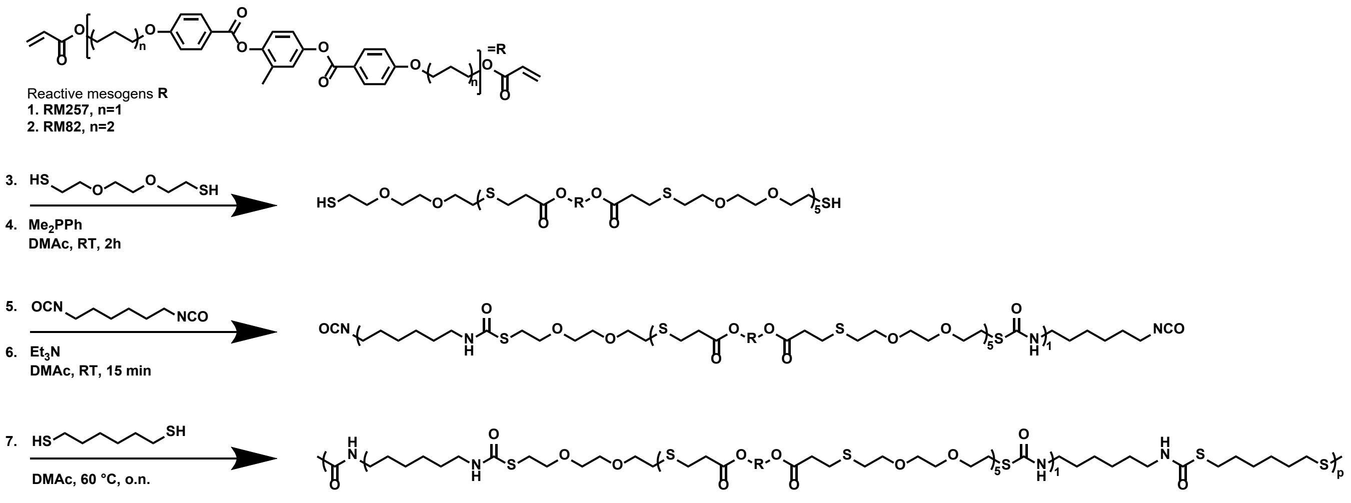

Figure S1. The synthesis of segmented PTU, using sequential addition reactions with reactive mesogens **1** (n=1, RM257) and **2** (n=2, RM82).

Table S1. Compounds and molar ratios used for the preparation of the LC thermoplastic PTU.

	Compound	Molar ratio (-)
**Diacrylate**	RM257 (**1**)	1
	RM82 (**2**)	1
**Dithiol**	EDDET (**3**)	2.4
**Phosphine catalyst**	DMPP (**4**)	0.012 (0.1 wt%)
**Diisocyanates**	HMDI (**5**)	2
**Dithiol**	HDT (**7**)	1
**Amine catalyst**	Triethylamine (**6**)	0.047 (0.1 wt%)

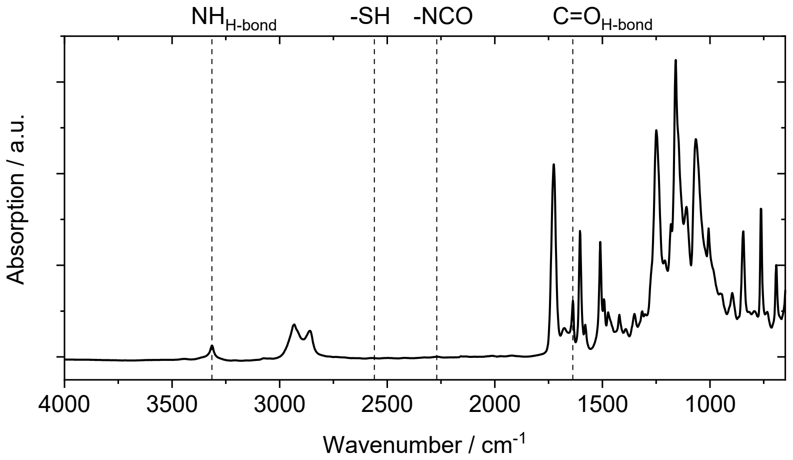

Figure S2. FTIR spectrum of the crude PTU. The dashed lines indicate the absence of thiol and isocyanate stretching vibrations at 2560 and 2270 cm^{-1}, respectively, and the presence of hydrogen-bonded TU amine and carbonyl stretching vibrations at 3315 and 1638 cm^{-1}, respectively.

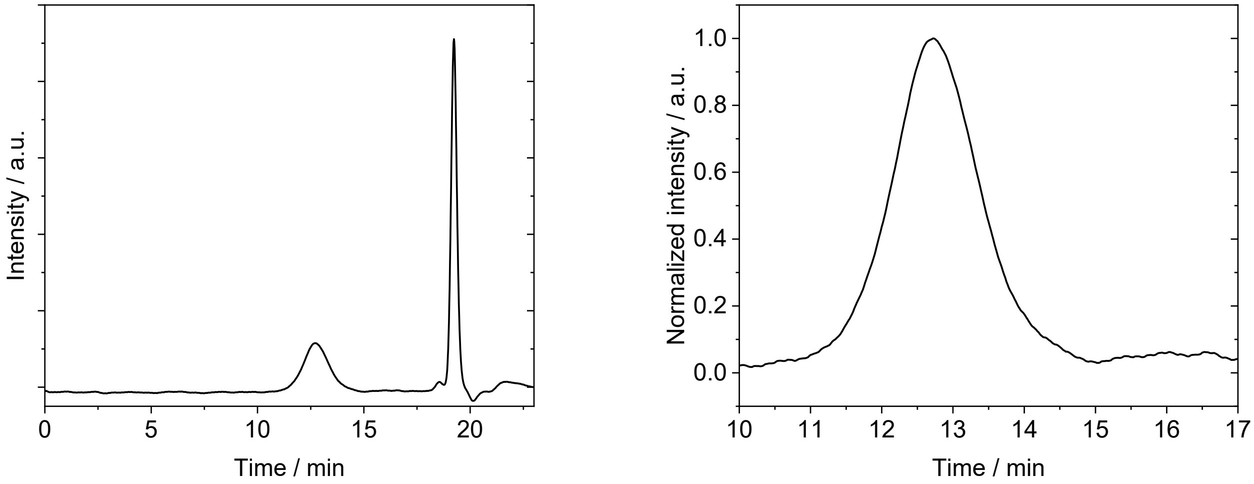

Figure S3. GPC data of the crude PTU (M_n = 72968 g mol^{-1} and polydispersity $Đ$ = 1.90) showing the full (left) and zoomed-in (right) chromatogram. The flow marker (toluene) is observed around 19 min.

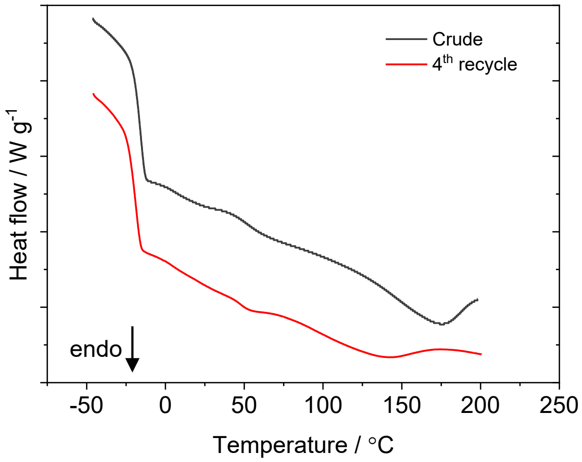

Figure S4. DSC thermographs of the crude material and printed 4th recycle actuator, showing the second heating run. The glass transition temperature of the soft LC segments is nearly unaffected upon recycling.

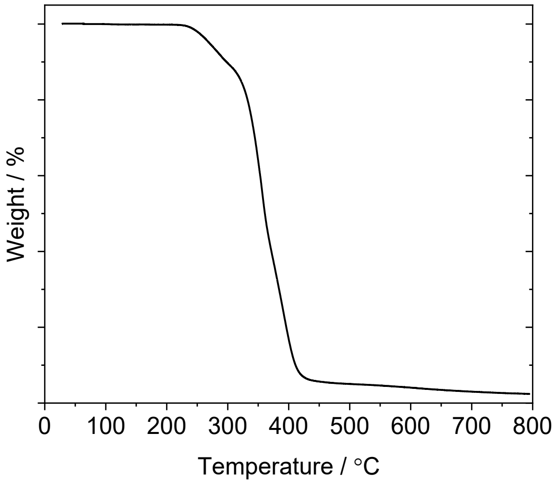

Figure S5. TGA profile of the crude PTU, showing two transitions corresponding to the degradation of TU and ester moieties.

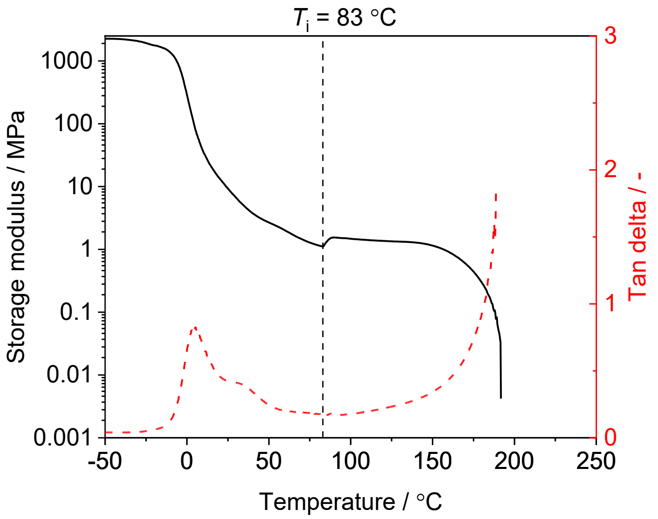

Figure S6. DMA storage modulus (E') and loss tangent (tan δ) of the compression-molded thermoplastic PTU as a function of temperature, with the T_i indicated by the black, dashed line. Above the glass transition temperature, a shoulder is observed around 30 °C arising from the increased order of the LC soft segment chains during heating.

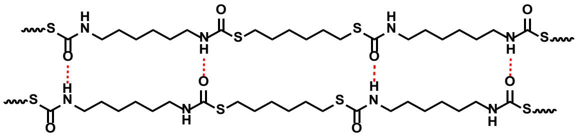

Figure S7. Molecular representation of the hydrogen-bonded TU segments.

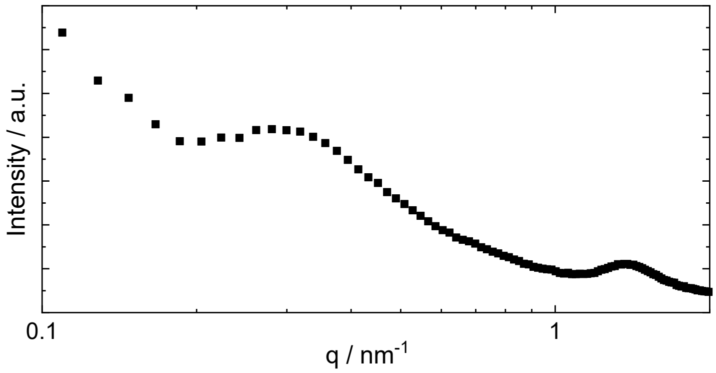

Figure S8. 1D MAXS diffractogram of the crude material showing a peak in the small-angle region at q = 0.29 nm^{-1} arising from the interdomain spacing between the microphase-separated domains. Additionally, a peak is observed at q = 1.39 nm^{-1} due to the spacing between the soft segment chains.

Table S2. Printing parameters used for DIW the thermoplastic PTU LCE.

Parameter	Set value
Printing Temperature (°C)	200
Bed Temperature (°C)	30
Nozzle diameter (mm)	0.3
Offset distance (mm)	0.2
Printing speed (mm s^{-1})	5
Line spacing (μm)	380

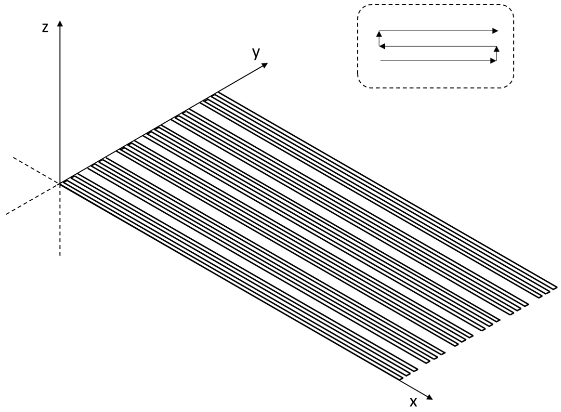

Figure S9. Schematic of the programmed printing path designed to make six actuator stripes (35×2 mm^2).

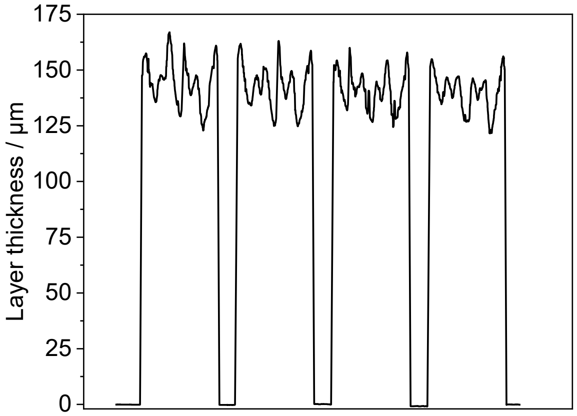

Figure S10. Layer thickness profile of four printed free-standing LCE actuators as obtained with an optical profiling system.

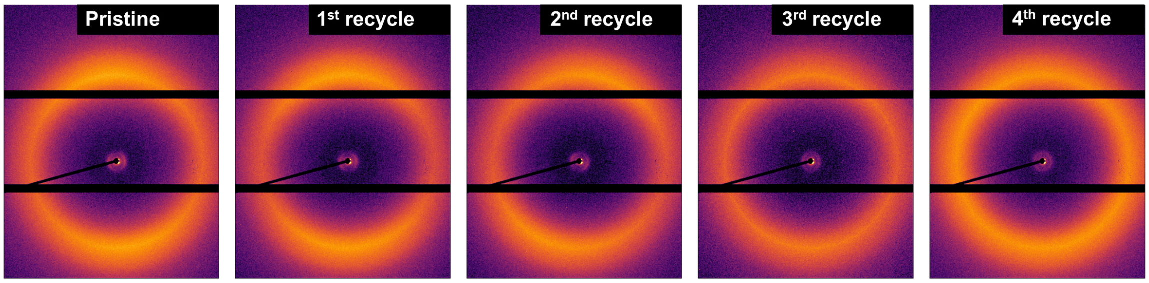

Figure S11. 2D WAXS diffractograms of the pristine – 4th recycle (left-to-right) printed PTU actuators. The molecular director of the aligned materials is horizontal.

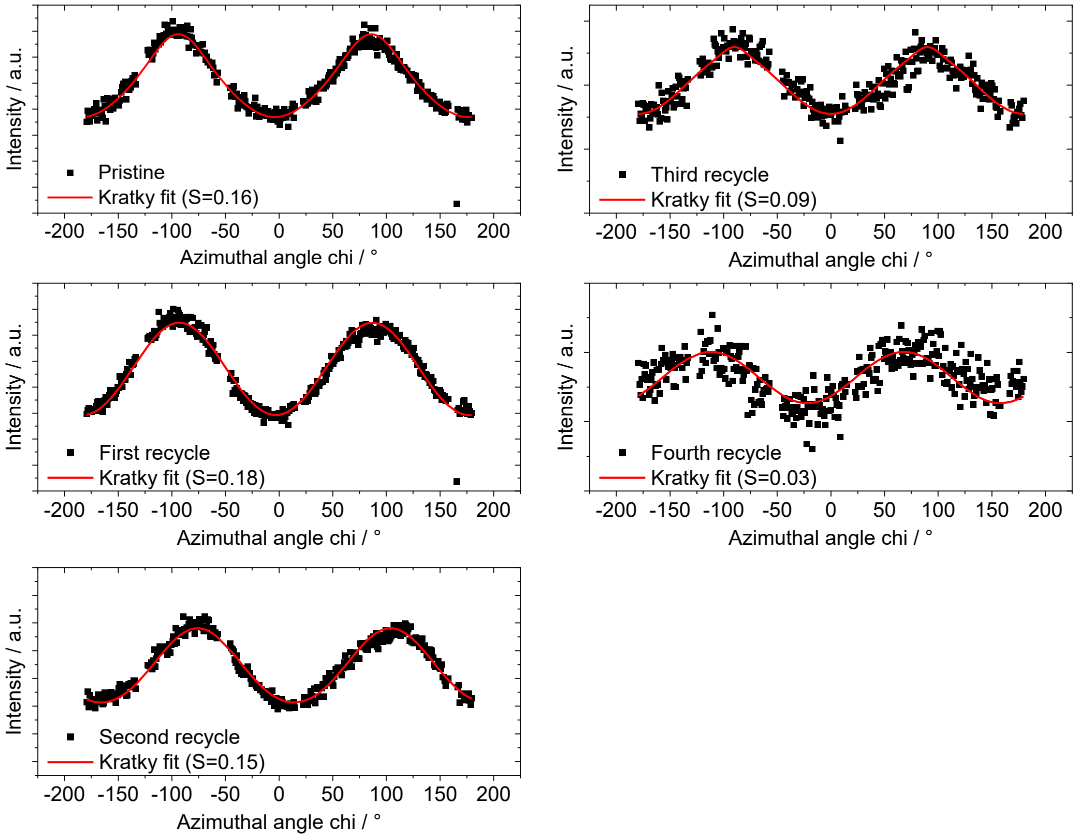

Figure S12. Azimuthal profiles of the 2D WAXS patterns at q = 14.4-14.6 nm^{-1} for the printing cycles. The order parameter is obtained by fitting a Kratky function over the azimuthal angle with a custom-made script.

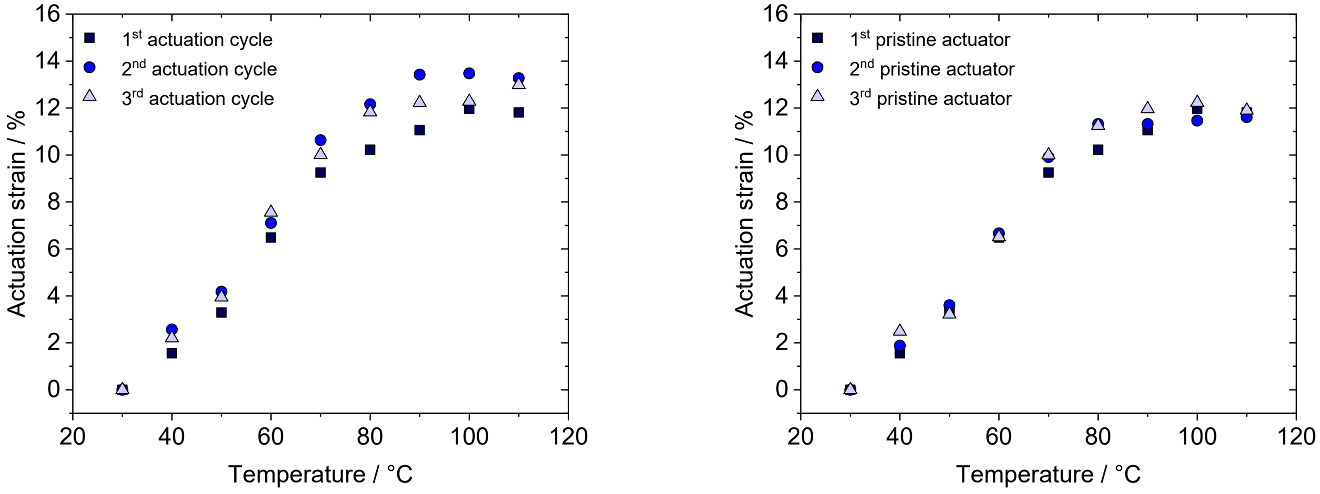

Figure S13. The actuation strain of the printed pristine actuator corresponding to Figures 2b and 2c showing three actuation cycles (left). The actuation strain of three different printed pristine actuators (right).

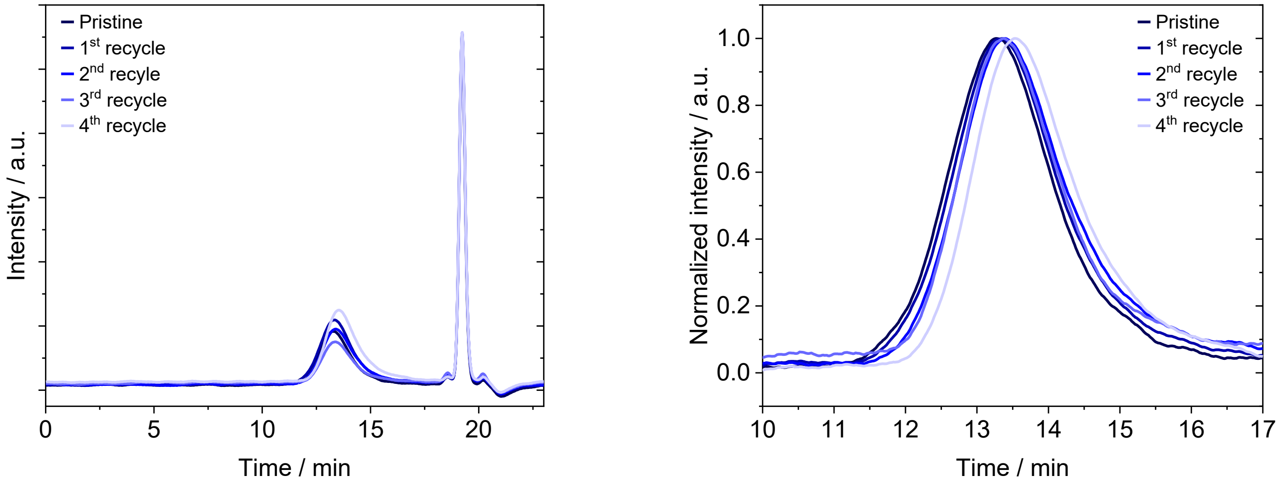

Figure S14. GPC data of the pristine and recycled printed PTU LCE actuators showing the full (left) and zoomed-in (right) chromatogram. The peak maximum shifts to higher retention times with an increasing number of printing (re)cycles. The flow marker (toluene) is observed around 19 min.

Table S3. Number average molar mass (M_n) and polydispersity ($Đ$) of the thermoplastic PTUs of the crude material and different printing cycles from the GPC results.

**PTU**	**M_n (g mol^{-1})**	**$Đ$**
**Crude**	72968	1.90
**Pristine**	39371	2.00
**1st recycle**	30962	2.17
**2nd recycle**	29844	1.98
**3rd recycle**	27857	1.96
**4th recycle**	21183	2.06

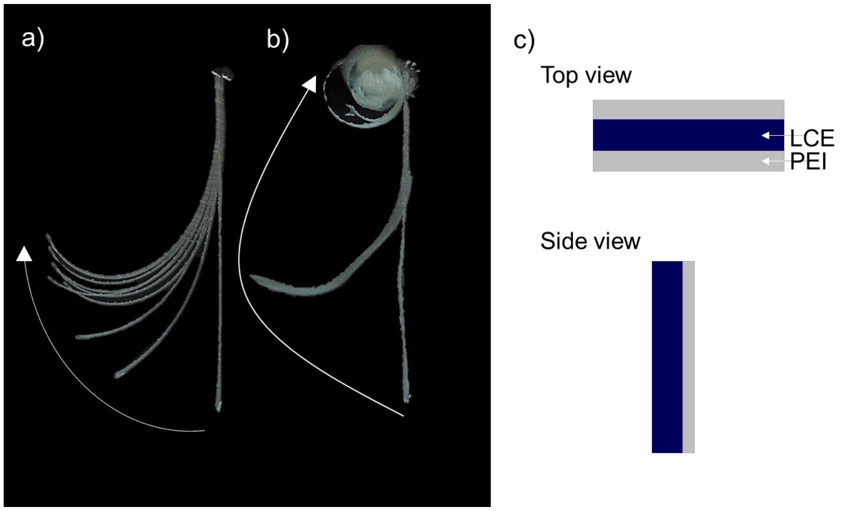

Figure S15. Stacked images of the patterned actuators (25 × 2 mm^2, 40% coverage, and ~140 μm of LCE) printed on a) 10 μm and b) 5 μm PEI substrates (25 × 5 mm^2) displaying an upward bending motion and a tight roll upon heating, respectively. The accompanying curling motion of the 5 μm PEI substrate in b) is observed since the film thickness is thinner. (c) Schematic representation of the top and side view of an LCE (blue) patterned PEI film (grey).

Supporting Video Captions

Video S1. Printing process of uniaxially aligned thermoplastic LCE strips. Playback rate: 8× (MP4).

References

(1) Sims, M. T.; Abbott, L. C.; Richardson, R. M.; Goodby, J. W.; Moore, J. N. Considerations in the Determination of Orientational Order Parameters from X-Ray Scattering Experiments. *Liq. Cryst.* **2019**, *46* (1), 11–24.

(2) del Pozo Puig, M., Sol, J. A. H. P., van Uden, S. H. P., Peeketi, A. R., Lugger, S. J. D., Annabattula, R. K., Schenning, A. P. H. J., Debije, M. G. Patterned Actuators via Direct Ink Writing of Liquid Crystals. *ACS Appl. Mater. Interfaces* **2021**, *13* (49), 59381–59391.
